# The Specific Low-Interference dsDNA Copper Nanoclusters for Visual Fluorescent Detection and Quantification of the *EGFR* L858R Point Mutation in Whole Single-Tube Magnetic Purification System

**DOI:** 10.1021/acs.analchem.6c00089

**Published:** 2026-04-27

**Authors:** Ke-Peng Lai, Ravery Sebuyoya, Kung-Hung Lin, Hwang-Shang Kou, Chun-Chi Wang

**Affiliations:** † School of Pharmacy, College of Pharmacy, Kaohsiung Medical University, Kaohsiung, 807, Taiwan; ‡ Department of Surgery, Zuoying Armed Forces General Hospital, Kaohsiung, 813, Taiwan; § Department of Family Medicine, Zuoying Armed Forces General Hospital, Kaohsiung, 813, Taiwan; ∥ Department of Medical Research, Kaohsiung Medical University Hospital, Kaohsiung, 807, Taiwan; ⊥ Drug Development and Value Creation Research Center, Kaohsiung Medical University, Kaohsiung, 807, Taiwan

## Abstract

Existing fluorescence-based techniques for single-nucleotide variation detection are limited by nonspecific fluorescence interference and complex analytical workflows. To address these challenges, we developed a single-tube fluorescence detection strategy integrating restriction fragment length polymorphism with poly-AAT–templated copper nanoclusters. Owing to the significantly higher synthesis efficiency of copper nanoclusters on AT-rich sequences than on random DNA templates, fluorescence interference from residual genomic DNA and nonspecific amplification products was effectively suppressed. As a result, strong fluorescence emission was generated predominantly from double-stranded poly-AAT, enabling reliable visual discrimination of single-nucleotide variation ratios under UV illumination. Biotin-labeled primers combined with streptavidin-coated magnetic beads enabled efficient separation of digested DNA fragments, eliminating the need for electrophoresis and further simplifying the workflow. The entire analytical procedure—including PCR amplification, enzymatic digestion, magnetic separation, and fluorescence measurement—was completed within a single tube, highlighting its suitability for automation and integration into microfluidic platforms. The method was validated for detection of the *EGFR* L858R mutation in patients with nonsmall cell lung cancer, yielding an excellent linear calibration curve (r = 0.9981), recovery rates of 95–110%, and a detection limit of 2.33%. These results demonstrate sensitivity comparable to that of commercial qPCR and next-generation sequencing, while offering advantages in instrument cost, simplicity, and analytical specificity.

## Graphical abstract

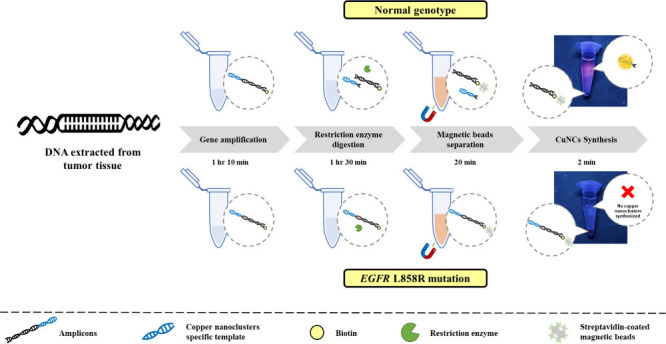

Fluorophores such as organic dyes are recognized for their robustness and strong fluorescence emission, which are critical for applications in diagnostic assays. However, previous studies have reported that fluorophores can increase background noise and interfere with primer annealing when attached to the 5′ end of primers.^1^ To address these limitations, DNA-templated fluorescent metal nanoparticles have attracted increasing attention in biomedical and biosensing applications owing to their small size, low toxicity, and excellent biocompatibility.^2,3^ Their distinctive optical, electronic, and catalytic properties have also been exploited in various analytical assays.^4^ Given their nanosized structure and the presence of numerous functional groups, DNA provides an ideal scaffold for the synthesis of metal nanoclusters.^5,6^ Among the various DNA-templated metal nanoclusters, copper nanoclusters exhibit notable advantages, including cost-effectiveness, rapid synthesis achievable at room temperature within minutes, and ease of operation.^7–9^ Consequently, copper nanoclusters (CuNCs) have been extensively employed in biosensing applications.^10,11^ For example, research has demonstrated the utility of copper nanoclusters in the detection of various analytes, including microRNAs, single-nucleotide variations (SNVs), and deletions or duplications associated with Duchenne muscular dystrophy.^1,12–14^

Lung cancer is among the leading causes of cancer-related mortality in developed countries, which includes small cell lung cancer (SCLC) and nonsmall cell lung cancer (NSCLC). Notably, NSCLC accounts for approximately 85% of lung cancer cases.^15^ Several oncogenes have been identified as contributors to the development of NSCLC, including *EGFR*, *KRAS*, *BRAF*, and *HER2/ERBB2*, which are activated through missense mutations, insertions, or deletions.^16^ The L858R single-nucleotide substitution (c.2573 *T* > G) in exon 21 of the *EGFR* gene has been reported in approximately 47.9% of NSCLC patients.^17,18^ Patients harboring the L858R mutation show increased sensitivity to EGFR tyrosine kinase inhibitors.^19^ The U.S. Food and Drug Administration has approved Osimertinib as a first-line treatment for NSCLC patients with *EGFR* exon 19 deletions or exon 21 L858R mutations. Therefore, the rapid and straightforward detection of the L858R mutation is imperative for NSCLC patients to enable timely and precise treatment decisions.

The detection of single-nucleotide variations (SNVs) remains challenging in genetic research because of their high sequence similarity to wild-type DNA. Various methodologies have been developed for the detection of SNVs, including DNA sequencing, allele-specific quantitative polymerase chain reaction (qPCR), PCR-capillary electrophoresis, melting curve analysis, PCR-restriction fragment length polymorphism (PCR-RFLP), and PCR-single strand conformation polymorphism (PCR-SSCP).^20–24^ Recent studies have developed advanced strategies to improve the detection of low variant allele frequencies or hard-to-distinguish mutations. These include long blocker displacement amplification coupled with qPCR for enriching low-frequency mutation hotspots,^25^ high-energy penalty SNV detection using an engineered CRISPR/Cas12a system to improve SNV recognition in wobble or GC-rich regions,^26^ and modified unit-mediated strand displacement reaction for rapid detection of SNVs in double-stranded DNA without denaturation.^27^ However, these techniques often rely on sophisticated instrumentation, precise operation or expensive reagents. Therefore, we aimed to develop a straightforward, cost-effective, and rapid analytical technique for SNVs detection.

In this study, we developed a single-tube, label-free analytical assay based on copper nanoclusters for quantifying the L858R point mutation in exon 21 of the *EGFR* gene in NSCLC patients. The primers employed in this study were specifically designed to facilitate the *in situ* synthesis of copper nanoclusters, which generate fluorescence visible to the naked eye. This method requires only a thermocycler and a fluorescence spectrometer to detect and quantify the L858R mutation in actual NSCLC patient samples. The entire reaction was conducted within a single tube. We anticipated that this method could serve as a preliminary diagnostic tool for NSCLC patients, to facilitate more precise therapeutic interventions. Furthermore, this analytical approach can be readily adapted for detecting various SNVs through the use of appropriate restriction enzymes.

## Experimental Section

### Chemicals and Reagents

The primers and templates listed in Table S1 were supplied by MDBio, Inc., situated in Taiwan. The PCR kit, which included a 10× PCR buffer, Taq polymerase (5 U/μL), and deoxynucleotide triphosphates (dNTPs) at a concentration of 2.5 mM, was acquired from Takara Biotechnology in Japan. Streptavidin magnetic beads (4 mg/mL; containing 0.1% BSA, 0.05% Tween 20, 0.05% NaN_3_, and 1× PBS at pH 7.4), along with the restriction enzyme MscI (5,000 U/mL) and the 10× CutSmart buffer solution (comprising 50 mM potassium acetate, 20 mM tris-acetate, 10 mM magnesium acetate, and 100 μg/mL BSA at pH 7.9), were obtained from New England Biolabs in the USA. 5× TBE buffer was sourced from Protech Technology Enterprise (Taipei, Taiwan), while the 100-bp DNA ladder was provided by Antec Bioscience Inc. (Taipei, Taiwan). YO-PRO-1 iodide was obtained from Molecular Probes (Invitrogen Detection Technologies, USA). 3-Morpholinopropane-1-sulfonic acid (MOPS), copper sulfate, sodium ascorbate and poly­(ethylene oxide) (PEO) with a molecular weight of approximately 8,000,000 were sourced from Sigma-Aldrich in the USA. Sodium chloride (NaCl) and sodium hydroxide (NaOH) were purchased from E. Merck in Germany. Double distilled water, produced by the Milli-Q water system (Millipore, USA), was utilized throughout the experimental procedures.

### Polymerase Chain Reaction

All polymerase chain reactions were conducted using a thermocycler (Biometra T Professional, Germany). The PCR reaction mixture (50 μL total) contained 5 μL of 10× PCR buffer, 200 μM each of dATP, dTTP, dCTP, and dGTP, 0.1 μM of both forward and reverse primers, and 50 ng of template DNA. After thorough mixing, the solution was subjected to an initial denaturation step at 95 °C for 3 min. This was succeeded by 35 cycles consisting of denaturation at 96 °C for 15 s, annealing at 56 °C for 15 s, and extension at 72 °C for 30 s, followed by a final extension at 72 °C for 10 min.

### Capillary Gel Electrophoresis

Capillary gel electrophoresis (CGE) was employed to validate the PCR results using the P/ACE MDQ Capillary Electrophoresis System (Beckman, USA). A coated fused-silica capillary (Agilent Technologies, USA) with an internal diameter of 100 μm and a total length of 40 cm (30 cm to the detector) was utilized. The capillary was filled with a gel buffer composed of 1% (w/v) PEO in 2× TBE, supplemented with 1 μL of YO-PRO-1 for DNA staining. Prior to analysis, all PCR products were diluted 1,000-fold. Sample injection was conducted electrokinetically at −8 kV for 5 s, followed by separation at a constant voltage of −10 kV for 20 min. The temperature was rigorously maintained at 25 °C throughout the procedure. A laser-induced fluorescence (LIF) detector, operating at an excitation wavelength of 488 nm and an emission wavelength of 520 nm, was employed for real-time monitoring of the separation process. Fragment sizes were determined by comparison with a 100-bp DNA ladder.

### MscI Restriction Enzyme Digestion and Streptavidin-Coated Magnetic Beads Separation

The PCR products were subsequently subjected to treatment with 50 μL of an MscI restriction enzyme solution, which contained different amounts of MscI (0, 1.25, 2.5, 3.75, and 5 units) within CutSmart buffer (composed of 10 mM potassium acetate, 4 mM Tris-acetate, 2 mM magnesium acetate, 2 μg recombinant albumin, pH 7.9). The digestion reactions were conducted in a Biometra T Professional thermocycler (Germany) at 37 °C for 0, 30, 60, 90, and 120 min, followed by enzyme inactivation at 80 °C for 20 min. Following the digestion process, varying amounts of streptavidin-coated magnetic beads (0, 20, 40, 60, and 80 μg) were introduced into the digested samples. The resulting mixtures were then incubated at room temperature for intervals of 0, 5, 10, 15, and 20 min prior to magnetic separation. After a magnetic separation period of 2 min, the supernatant was clear and used directly for subsequent analysis.

### Copper Nanoclusters Synthesis Optimization

In this study, copper nanoclusters were synthesized using a uniform length of 48-mer poly-AT, poly-AAT, poly-T, and randomly designed sequence at the 5′ end of the reverse primer to identify the most effective sequence for subsequent research (refer to Table S1). The incorporation of these sequence extensions facilitated a more efficient synthesis of copper nanoclusters. The outcomes were evaluated by measuring the relative fluorescence intensity, denoted as F–F_0_. Following the completion of the experimental procedures, a supernatant volume of 70 μL was treated with MOPS buffer (10 mM MOPS, 150 mM NaCl, pH 7.5). Subsequently, various concentrations of copper sulfate (0, 80, 160, 240, and 320 μM) were utilized to optimize the formation of copper nanoclusters, with sodium ascorbate (0, 1, 2, 3, and 4 mM) serving as the reducing agent, resulting in a total volume of 200 μL. The resulting mixture was incubated at room temperature for 3 min prior to measurement.

### Fluorescence Measurement

The fluorescence of the copper nanoclusters was measured using the F-4500 fluorescence spectrometer (Hitachi, Japan). The data acquisition mode was configured for luminescence, with a photomultiplier tube (PMT) voltage set at 700 V. Both the excitation and emission slits were adjusted to 5 nm. The excitation wavelength was set at 350 nm, while the emission wavelength was measured within the range of 500 to 650 nm. The scanning speed was set to 1200 nm/min. To account for the variable signal associated with copper nanoclusters, we activated the mean smoothing mode and specified a 50-point average to achieve a more accurate measurement of fluorescence intensity. Transmission electron microscopy (TEM) images of the copper nanoclusters were obtained using a JEOL JEM-3010 microscope.

### Quantification Assay and Real Samples Analysis

DNA samples were obtained from consenting individuals at Kaohsiung Medical University Chung Ho Hospital in Kaohsiung, Taiwan. The genomic DNA was extracted from the peripheral whole blood of patients using the ZEJU Genomic DNA extraction kit (ZEJU Co., Ltd., Taiwan) following the manufacturer’s instructions. These gDNA samples were utilized as a wild-type background matrix to simulate the cellular heterogeneity and variable tumor purity typical of clinical tissue biopsies. The optical density (OD) ratios at 260/280 for the extracted DNA samples ranged from 1.8 to 2.0, as measured by a U-2900 spectrophotometer (Hitachi, Japan). The final concentration of the DNA was adjusted to 50 μg/mL using double-distilled water prior to spiking with *EGFR* L858R reference standards to evaluate analytical performance.

In this study, we increased the PCR cycle count to 45 in order to achieve a consistent analytical effect in the genomic DNA samples. Following the optimization of the assay conditions, we evaluated various compositions of a 50 ng DNA template to construct a calibration plot. Specifically, a series of DNA mixtures were prepared with variant allele frequencies (VAFs) of 0%, 20%, 40%, 60%, 80%, and 100% by combining the L858R mutant template with the wild-type template. The total DNA quantity was consistently maintained at 50 ng per reaction to construct a standard curve. Subsequently, we analyzed DNA samples from ten different individuals and compared the results of our assay with those obtained through Sanger sequencing. To further validate the efficacy of our technique, we utilized the *EGFR* L858R Reference Standard at 50% (Horizon Discovery Ltd., UK) and the *EGFR* L858R Reference Standard at 100% (GeneCopoeia Inc., US), both of which were derived from genomic DNA extracted from human cell lines, to simulate DNA samples extracted from nonsmall cell lung cancer (NSCLC) tissues. To evaluate potential interference and the generation of false-positive results from adjacent mutations within the same exon, an additional test was conducted by directly analyzing 50 ng of a synthetic *EGFR* L861Q template. The resulting fluorescence signals were subsequently compared with those obtained from 50 ng of the wild-type and L858R mutation templates under optimized experimental conditions.

### Single-Tube Analysis and Visual Detection

In the single-tube analysis, the entire procedure is conducted within a single PCR tube. Initially, 50 μL of PCR product is mixed with 50 μL of the MscI restriction enzyme solution. Following the digestion process, 15 μL of SMBs is added, resulting in a total mixture volume of 115 μL. To this mixture, 160 μM of copper sulfate, 10 mM MOPS buffer (containing 150 mM NaCl, pH 7.5), and 3 mM sodium ascorbate are incorporated to achieve a final volume of 200 μL. Ultraviolet light at a wavelength of 365 nm is employed to excite the copper nanoclusters within the PCR tube.

## Results and Discussion

### Mechanism for Detecting Exon 21 (L858R) Point Mutation

The workflow for detecting the *EGFR* exon 21 L858R point mutation is illustrated in Figure 1, with all analytical steps performed within a single PCR tube. Genomic DNA extracted from tumor tissues of patients with nonsmall cell lung cancer was first amplified by PCR using specifically designed primers. The forward primer was biotinylated at the 5′ end, and a poly-AAT sequence was incorporated at the 5′ end of the reverse primer. This primer design enabled the generation of PCR amplicons that could be selectively captured by streptavidin-coated magnetic beads while simultaneously carrying a double-stranded poly-AAT region that served as an efficient template for copper nanocluster formation. Following amplification, the restriction enzyme MscI was employed to selectively digest the wild-type *EGFR* sequence, whereas the L858R mutant sequence remained intact due to the loss of the recognition site. After enzymatic digestion, streptavidin-coated magnetic beads were introduced to bind the biotinylated amplicons via streptavidin–biotin interactions, enabling magnetic separation of the DNA fragments. In wild-type samples, MscI digestion cleaved the amplicons between the biotin-labeled region and the poly-AAT–containing segment, generating DNA fragments that lacked the biotin label but retained the double-stranded poly-AAT region. These fragments remained in the supernatant after magnetic separation, where efficient copper nanocluster formation produced a strong fluorescence signal. In contrast, mutant amplicons lacked the MscI recognition site and therefore remained intact, allowing the biotinylated, poly-AAT–containing products to be captured and removed from the supernatant using streptavidin-coated magnetic beads. As a result, almost no poly-AAT–containing DNA remained in the supernatant, leading to significantly reduced copper nanocluster formation and a weak fluorescence signal. This marked difference in fluorescence intensity enabled effective discrimination between wild-type and *EGFR* L858R mutant genotypes.

**Figure 1:**
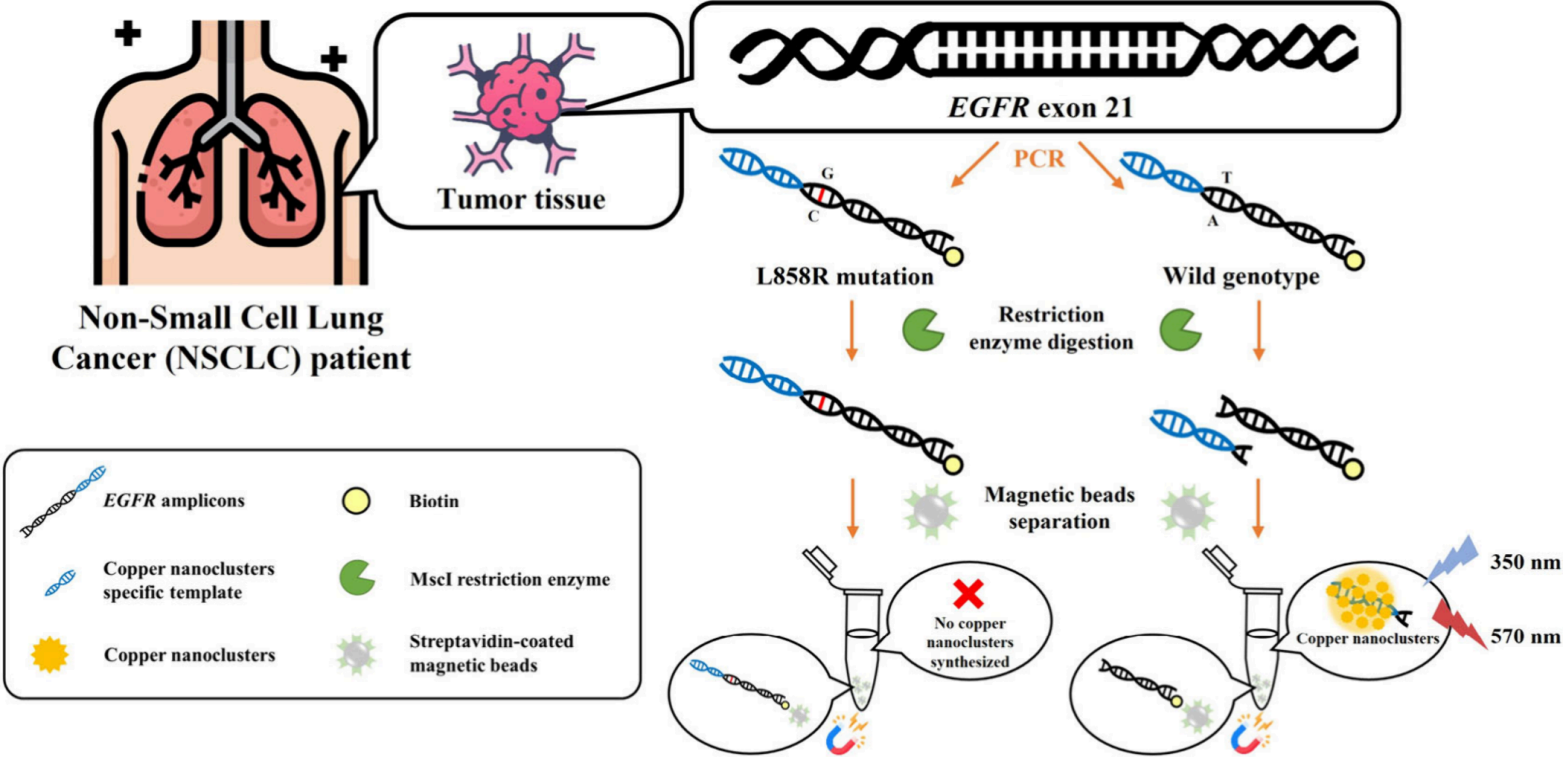
Mechanism of detecting L858R mutation in nonsmall cell lung cancer (NSCLC) patient.

### Analysis Results of Different Primer Sequences

The primary objective of this study is to address the challenges associated with synthesizing copper nanoclusters using DNA as a template. The poly-AT sequence had been identified as an effective sequence for copper nanoclusters synthesis. However, single-stranded poly-AT sequence perform poorly in PCR applications due to self-complementarity.^28^ The fundamental cause of this phenomenon is that, even in the absence of amplification, primers labeled with poly-AT can undergo self-annealing, leading to the formation of double-stranded poly-AT templates. This occurrence significantly increases fluorescent interference in blank samples. Based on our previous findings, we replaced the poly-AT sequence at the 5′ end of the primer with a poly-AAT sequence. This modification reduces interference in negative samples by introducing periodic mismatches during primer self-annealing, thereby reducing the synthesis efficiency of copper nanoclusters.^29^ To validate this hypothesis, a comparative analysis of the designed primers (Table S1) was performed at the beginning of our research (Figure 2A). Although the *EGFR*-48AT primer produced the highest fluorescence intensity, it also caused significant fluorescent interference in L858R samples (Figure S1A). This interference is likely attributed to self-annealed poly-AT double-stranded sequence residues in the supernatant that could not be removed by streptavidin-coated magnetic beads. In contrast, the *EGFR*-48AAT primer generated moderate fluorescence intensity in wild-type samples and markedly reduced interference in the L858R samples (Figure S1B). Although self-annealed poly-AAT sequences may also persist in the supernatant, their fluorescence interference is significantly diminished due to the presence of periodic mismatches within the sequence. Comparatively, the fluorescence intensities obtained using the *EGFR*-48T primer and *EGFR*-48Random primers were lower than those produced by the *EGFR*-48AT and *EGFR*-48AAT primers (Figure S1C, D). Therefore, the poly-AAT extended primer was selected and consistently employed in subsequent experiments.

**Figure 2:**
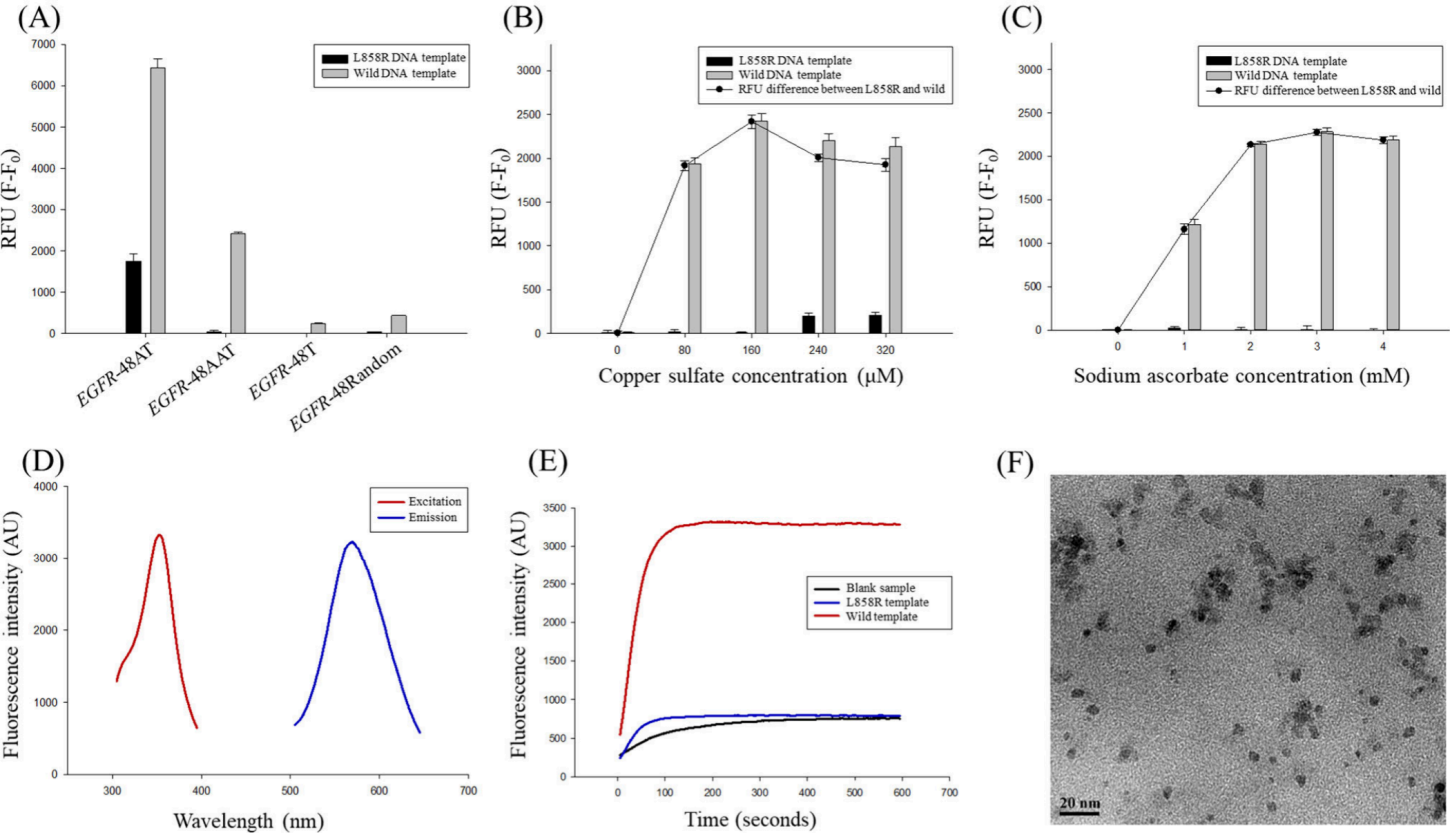
Different conditions of synthesizing and detecting copper nanoclusters throughout the assay. (A) Utilization of different primer sequences for the synthesis of copper nanoclusters in the supernatant (*n* = 3). (B) Varying copper sulfate concentrations for synthesizing copper nanoclusters on the poly-AAT sequence (*n* = 3). (C) Different sodium ascorbate concentrations for synthesizing copper nanoclusters on the poly-AAT sequence (*n* = 3). (D) Fluorescence excitation (red line) and emission (blue line) wavelength scans for poly-AAT templated copper nanoclusters. (E) Time scan of fluorescence intensity for blank (black line), L858R (blue line), and wild (red line) samples. (F) TEM spectra of copper nanoclusters from the wild sample supernatant. RFU = F – F_0_, where F is the fluorescence of L858R and wild DNA templates, and F_0_ is the fluorescence of the blank sample.

### Optimization for Copper Nanoclusters Synthesis

Copper sulfate and sodium ascorbate are the reagents most commonly used in the synthesis of DNA-templated copper nanoclusters.^30–32^ In this process, copper sulfate serves as the source of copper ions, whereas sodium ascorbate acts as the reducing agent. The concentration of copper ions is a critical factor in the synthesis of copper nanoclusters; therefore, we examined various concentrations of copper sulfate. The results indicated that a concentration of 160 μM of copper sulfate was optimal for the synthesis of copper nanoclusters on the poly-AAT labeled amplicons and was consistently employed throughout the study (Figure 2B). Regarding sodium ascorbate, the relative fluorescence unit (RFU) difference increased and reached saturation at 3 mM. Following this point, a slight decrease in fluorescence intensity was observed in wild-type samples (Figure 2C). Previous study has shown that excess reducing agent decreases the fluorescence intensity of copper nanoclusters, which is consistent with our findings.^33^ Therefore, 3 mM of sodium ascorbate was selected as the optimized concentration for synthesizing poly-AAT-templated copper nanoclusters.

### Properties of Poly-AAT-Templated Copper Nanoclusters

The optimized poly-AAT templated copper nanoclusters exhibited optimal excitation and emission wavelengths at 350 and 570 nm, respectively (Figure 2D). The fluorescence intensity of the synthesized copper nanoclusters across all samples demonstrated stability over a duration of 10 min, which meets the minimum requirement for analytical detection (Figure 2E). In comparison, previous studies had demonstrated that DNA-templated copper nanoclusters generally exhibit a gradual decrease in fluorescence intensity, ultimately leading to complete quenching.^5,34^ The comparatively increased stability observed in our system can be attributed to the presence of stabilizing agents for copper nanoclusters, such as glycerol or saccharides, within the residual PCR and restriction enzyme reagents.^30^ The morphology of the synthesized copper nanoclusters was further characterized using transmission electron microscopy, with diameters ranging from 2 to 4 nm (Figure 2F).

### Optimization of Streptavidin-Coated Magnetic Beads and MscI Restriction Enzyme Condition

Streptavidin magnetic beads (SMBs) were introduced to capture the biotinylated amplicons, enabling magnetic separation. Various amounts of SMBs were tested, and the resulting fluorescence intensities were analyzed (Figure 3A). The relative fluorescence units (RFU) difference between L858R mutant and wild-type samples increased with increasing amounts of SMBs. A total of 60 μg of SMBs was determined to be optimal for achieving signal saturation and was used consistently in subsequent experiments. Regarding incubation time, the RFU of the L858R samples decreased to nearly zero after 20 min (Figure 3B); therefore, an incubation time of 20 min was selected.

**Figure 3:**
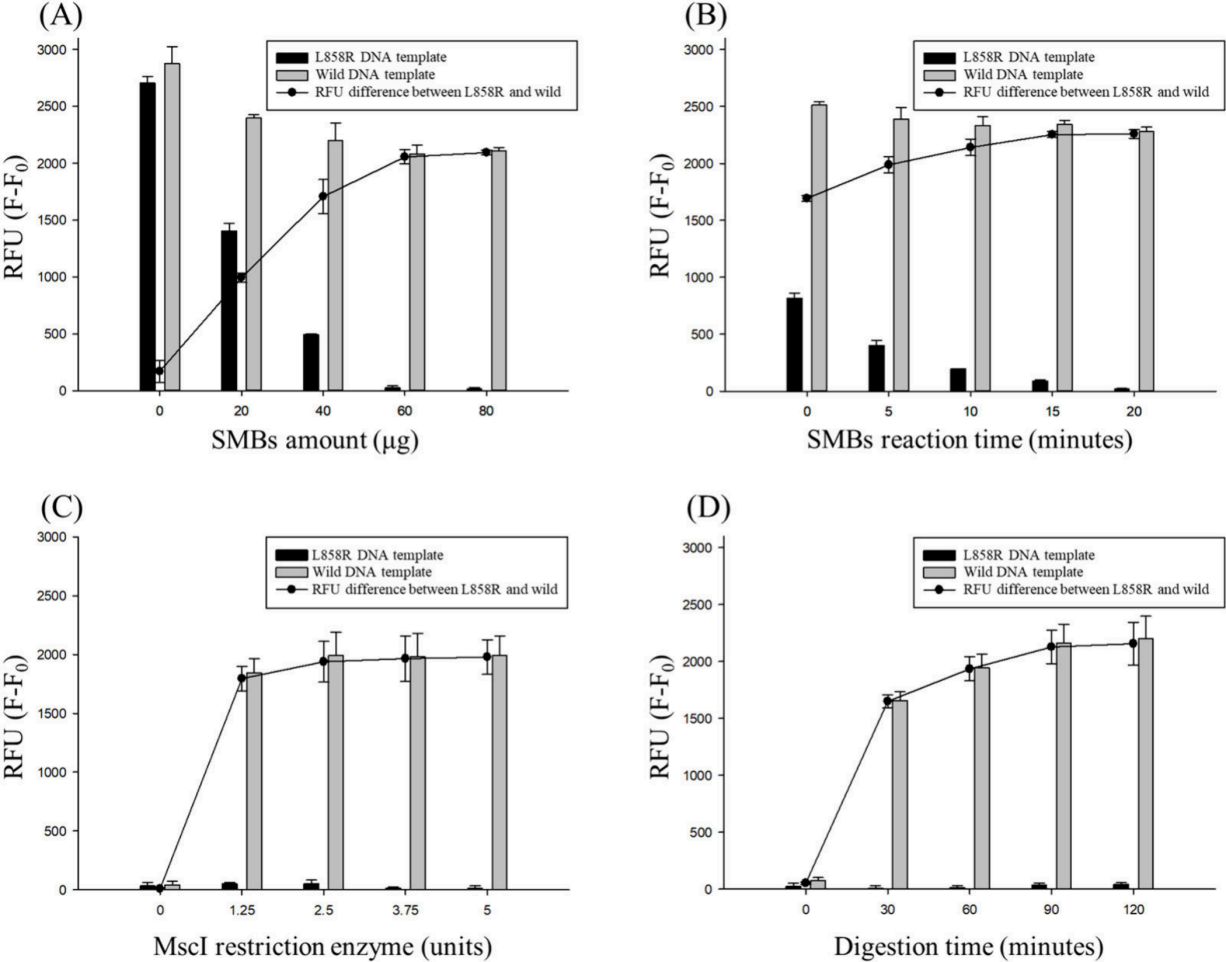
Optimization of the condition of SMBs and MscI restriction enzyme. (A) The amount of SMBs added after MscI digestion (*n* = 3). (B) The reaction time after adding SMBs to the digested amplicons (*n* = 3). (C) The amount of MscI restriction enzyme added to digest PCR amplicons (*n* = 3). (D) The reaction time for MscI restriction enzyme digestion (*n* = 3). RFU = F – F_0_. F: the fluorescence of L858R and wild DNA template. F_0_: the fluorescence of blank sample.

The MscI restriction enzyme specifically recognizes and cleaves the DNA sequence TGGCCA. In the present study, the wild-type amplicons possess this recognition site. Conversely, the L858R mutation involves a thymine-to-guanine substitution that abolishes the recognition site, thereby preventing enzymatic digestion. When comparing the results obtained with varying enzyme concentrations, 2.5 units of MscI were found to be sufficient for the analysis (Figure 3C). Regarding the reaction time for enzyme digestion, no significant increase in RFU difference was observed when the reaction time exceeded 90 min (Figure 3D). Further increases in enzyme concentration or digestion time could result in nonspecific cleavage.^35^ Therefore, 2.5 units of the MscI were used for 90 min of digestion, followed by incubation with 60 μg of SMBs for 20 min in subsequent experiments.

### Quantification and Application in Real Samples

The fluorescence results indicated that the amplification efficiency in genomic DNA samples was lower than expected. This may be caused by the suboptimal PCR amplification of the PCR kit used in this study and the extended poly-AAT sequence on the primers, which may have caused steric hindrance during the annealing process. To compensate for this, the PCR cycle number was increased to 45, which restored the fluorescence intensity of the amplification products (Figure S2A). The specificity of the PCR products was confirmed through capillary gel electrophoresis. The electropherograms obtained displayed a single, distinct peak with a migration time consistent with the 300 to 400 base pair range of the 100-bp DNA ladder, aligning precisely with the expected theoretical size of 316 base pairs. No secondary peaks or primer dimers were detected, even after increasing the PCR cycle number from 35 to 45 (Figure S2B).

The *EGFR* L858R mutation ratio refers to the proportion of mutant alleles among total *EGFR* gene copies in a sample. This ratio reflects tumor heterogeneity, as both wild-type and mutant DNA sequences may coexist due to variations in tumor cell populations and the presence of normal cells within the same tissue sample.^36,37^ Accordingly, a calibration curve was established to quantify the *EGFR* L858R mutation ratio in NSCLC patients. The mutation ratio was determined based on the measured fluorescence intensity using the linear regression equation *y* = −28.114*x* + 3358.2, where *y* represents the raw, unsubtracted fluorescence intensity and *x* represents the *EGFR* L858R mutation ratio (%). The calibration curve showed excellent linearity (r = 0.9981) between fluorescence intensity and the L858R mutation ratio (Figure 4A), with a limit of detection (LOD) of 2.33% and a limit of quantification (LOQ) of 11.23%. The fluorescence differences were visually distinguishable under UV light, allowing direct identification of mutation ratios by the naked eye (Figure 4B). This method relies on reverse fluorescence detection, which provides two distinct advantages. First, this method enables clear visual differentiation between mutation ratios of 0% and 20% under UV illumination, whereas traditional forward fluorescence produces signals that are barely visible to the naked eye. Second, the reverse design inherently prevents potential false positives caused by an abundant wild-type background. Since pure wild-type samples generate the maximum baseline signal, the presence of mutations results in a signal decrease. Therefore, the LOD at 2.33% serves as a definitive cutoff, with a positive mutation call requiring a significant signal drop below this threshold.

**Figure 4:**
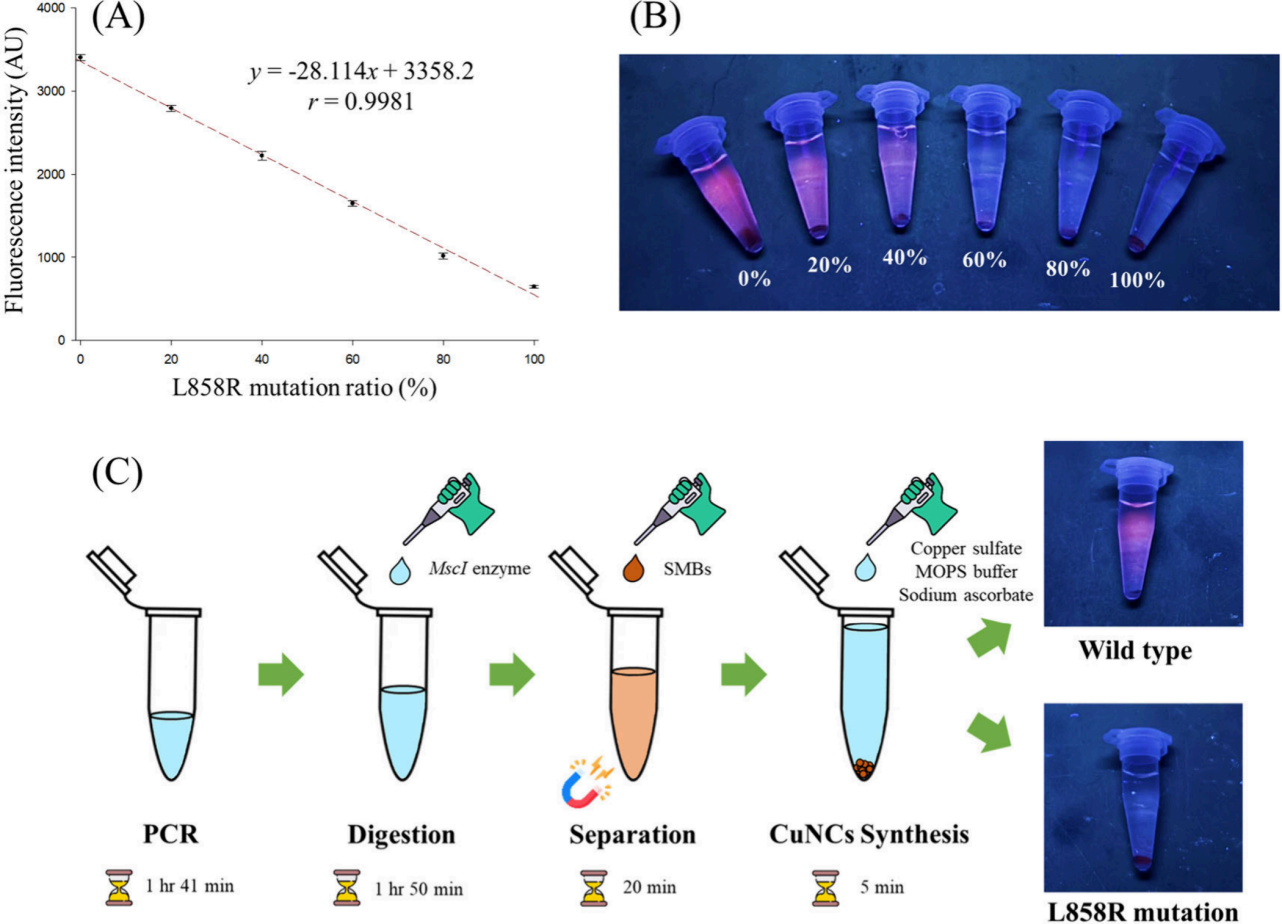
Quantification and analytical process of *EGFR* L858R mutation in real samples. (A) The calibration curve from 0 to 100% L858R mutation with optimized condition (*n* = 3). (B) Single-tube analysis image of 0, 20, 40, 60, 80, 100% L858R mutation (from left to right) obtained under 365 nm UV light. (C) The whole reaction process of our method completed within a single PCR tube.

Recovery studies using *EGFR* L858R reference standards spiked into the wild-type matrix confirmed high precision and accuracy, with recoveries ranging from 95% to 110% and relative standard deviations (RSD) below 5% (Table 1). The assay’s sensitivity (LOD = 2.33%) was comparable to that of commercial real-time PCR (qPCR) and approached that of next-generation sequencing (NGS) methods, which typically report LODs ranging from 1 to 5% for standard qPCR, and approximately 0.8–2% for NGS.^38,39^ Furthermore, the unspiked genomic DNA samples from ten individuals all exhibited fluorescence signals above the LOD threshold (Figure S3A), confirming their pure wild-type status and validating their suitability as a clean background matrix. These findings were further confirmed by Sanger sequencing (Figure S3B).

**Table 1:** Recovery and RSD of *EGFR* L858R Reference Standard and Spiked gDNA Samples (n = 3)

Genomic DNA sample	Recovery	RSD
*EGFR* L858R Reference Standard 50%	98.10%	1.98%
*EGFR* L858R Reference Standard mixed to 75%	97.20%	2.23%
*EGFR* L858R Reference Standard 100%	95.00%	4.65%
gDNA spiked with reference standard to 5% mutant	108.04%	1.56%
gDNA spiked with reference standard to 10% mutant	103.22%	1.10%

The assay’s specificity relies on the precise MscI restriction site at the L858R locus. Sequence analysis confirms that other mutations within exon 21 are exceedingly rare, and even the most notable one, L861Q, is located completely outside this recognition sequence.^40^ This was empirically verified by directly analyzing a synthetic *EGFR* L861Q template (Figure S4). The L861Q variant generated a high fluorescence intensity identical to the pure wild-type sequence. Conversely, only the target L858R mutation triggered a significant signal drop to the blank level, confirming the specificity of this assay and zero cross-reactivity.

### Single-Tube Analysis and the Advantages of This Assay

The entire analytical workflow described in this study can be conducted within a single PCR tube through a stepwise reagent-addition process (Figure 4C). Initially, the *EGFR* gene fragment was amplified by PCR within the tube. After amplification, the restriction enzyme was directly added to the reaction mixture to selectively recognize and cleave the amplified sequences. Subsequently, streptavidin-coated magnetic beads (SMBs) were introduced to separate the DNA fragments, followed by the *in situ* synthesis of copper nanoclusters (CuNCs) in the supernatant. The resulting fluorescence intensity was used to quantify the proportion of the *EGFR* L858R mutation.

Although the tube lid needs to be opened intermittently for reagent addition, the analytical process does not require transferring samples between containers, thereby minimizing the risk of contamination and simplifying the overall procedure. This sequential reagent-addition strategy preserves the integrity of the single-tube workflow, ensuring that amplification, digestion, separation, and fluorescence detection all occur within the same reaction tube. The design not only improves operational convenience and reproducibility but also significantly minimizes sample loss commonly associated with conventional multistep handling procedures. Furthermore, by integrating amplification and fluorescence quantification within a single-tube format, this method reduces reliance on sophisticated instrumentation and simplifies single-nucleotide variant analysis. The streamlined workflow and visual readout highlight its strong potential for integration into automated microfluidic platforms.

### Comparative Analysis of Methods and the Application of This Assay

Compared with assays that rely on fluorophore-labeled primers or DNA-binding dyes for signal generation, the poly-AAT–templated copper nanocluster strategy offers several advantages (Figure S5).^41,42^ The use of fluorophore-labeled primers provides high specificity, as only fluorophore-tagged fragments emit fluorescence, while residual genomic DNA (gDNA) and other nontarget products do not produce such signals. However, unreacted primers emit strong fluorescence, interfering with signal interpretation and necessitating PCR purification before restriction enzyme digestion (Figure S5A). In contrast, when DNA-binding dyes are used, single-stranded primers do not generate fluorescence prior to amplification, thereby eliminating the need for purification. However, during fluorescence detection in the supernatant, residual genomic DNA and nonspecific products can also bind to the dye and produce strong fluorescence signals, resulting in reduced detection specificity (Figure S5B).

The methodology utilizing poly-AAT-templated copper nanoclusters provides both high specificity and a simplified workflow without purification. The poly-AAT-labeled self-annealed primers produce a minimal quantity of copper nanoclusters, thus eliminating the need for purification. The efficiency of copper nanocluster synthesis in the supernatant is significantly enhanced in the presence of double-stranded poly-AAT sequences. Due to the high copper nanoclusters yield synthesized from the poly-AAT sequence, the small contribution from residual gDNA and other nonspecific products exerts a negligible influence on detection (Figure S5C). This assertion is supported by previous findings showing that double-stranded AT-rich sequences possess significantly higher synthesis efficiency than non-AT-rich sequences.^8^ To further validate this hypothesis, varying amounts of gDNA (50, 100, 150, and 200 ng) were spiked into the supernatant prior to copper nanocluster synthesis. As shown in Figure S6, the fluorescence intensity of the synthesized copper nanoclusters showed only a slight increase even when large amounts of gDNA were spiked. These results provide strong evidence that random-sequence DNA has minimal impact on our detection system. Based on this comparative analysis, the poly-AAT–templated copper nanocluster strategy achieves detection specificity comparable to fluorophore-based approaches. In addition, the low background fluorescence of poly-AAT–labeled primers prior to amplification eliminates the need for purification, enabling the entire assay to be performed within a single tube.

### Generalization and Assay Design Considerations

The analytical platform can be adapted to detect other point mutations by redesigning specific primers and selecting suitable restriction enzymes. To achieve this, primer design must ensure the amplicon contains a single restriction site overlapping the target mutation to successfully distinguish wild-type from mutant alleles. Furthermore, a second cleavage site within the amplicon must be strictly avoided to maintain assay specificity. Regarding amplicon length, the length itself does not significantly impact the final detection signal. This is primarily because the fluorescence signal is highly specific to the copper nanoclusters templated by the newly synthesized poly-AAT sequence. Since random double-stranded DNA from PCR or enzymatic digestion produces negligible copper nanoclusters, amplicon or fragment length variations do not interfere with the readout. Consequently, this unique signal generation mechanism offers considerable flexibility in sequence design, enabling straightforward adaptation to various genetic targets.

## Conclusions

This study presents an integrated analytical strategy that combines single-nucleotide mutation detection, single-tube operation, and visual fluorescence readout—capabilities that are rarely achieved within a single platform. All reaction steps are completed in a single PCR tube, yielding results that are directly observable by the naked eye. The fluorescence signal decreases progressively with increasing proportions of the *EGFR* L858R mutation, enabling quantitative discrimination of mutation ratios. A detection limit of 2.33% was achieved, demonstrating performance comparable to that of commercial real-time PCR kits and next-generation sequencing methods. Owing to its operational simplicity and visual readout, this system shows strong potential for integration into microfluidic devices. Furthermore, when combined with appropriate restriction enzymes, the platform may be extended as a versatile approach for detecting diverse gene mutations.

## Supplementary Material



## Notes

The sequence of primers and template; Analysis results of fluorescent wavelength scans using four different primers; Fluorescent signal comparison between 35 and 45 cycles; Real sample analysis in ten different individuals; Analytical specificity using *EGFR* L861Q template; Comparing different methods for DNA fluorescence detection with this assay and Fluorescence intensity of poly-AAT-templated copper nanoclusters spiked with genomic DNA (PDF)

## Abbreviations

CuNCs: copper nanoclusters
EGFR: epidermal growth factor receptor
SNVs: single-nucleotide variations
SCLC: small cell lung cancer
NSCLC: nonsmall cell lung cancer
MOPS: 3-Morpholinopropane-1-sulfonic acid
CGE: capillary gel electrophoresis
TEM: transmission electron microscope
RFU: relative fluorescence unit
SMBs: streptavidin magnetic beads
qPCR: real-time PCR
NGS: next-generation sequencing
gDNA: genomic DNA

## References

[1] Chen C. A., Wang C. C., Jong Y. J., Wu S. M. (2015). Label-Free Fluorescent
Copper Nanoclusters for Genotyping of Deletion and Duplication of
Duchenne Muscular Dystrophy. Anal. Chem..

[2] Jiang X., Du B., Huang Y., Zheng J. (2018). Ultrasmall Noble Metal Nanoparticles:
Breakthroughs and Biomedical Implications. Nano
Today.

[3] Zhang L. B., Wang E. K. (2014). Metal nanoclusters: New fluorescent probes for sensors
and bioimaging. Nano Today.

[4] Chen X., Yang D., Tang Y., Miao P. (2018). DNA-templated copper
nanoparticles for voltammetric analysis of endonuclease activity. Analyst.

[5] Qing T., Zhang K., Qing Z., Wang X., Long C., Zhang P., Hu H., Feng B. (2019). Recent progress in
copper nanocluster-based fluorescent probing: a review. Mikrochim. Acta.

[6] Liu J. W. (2014). DNA-stabilized,
fluorescent, metal nanoclusters for biosensor development. TrAC, Trends Anal. Chem..

[7] Qing Z. H., He X. X., He D. G., Wang K. M., Xu F. Z., Qing T. P., Yang X. (2013). Poly­(thymine)-Templated
Selective
Formation of Fluorescent Copper Nanoparticles. Angew. Chem., Int. Ed. Engl..

[8] Rotaru A., Dutta S., Jentzsch E., Gothelf K., Mokhir A. (2010). Selective
dsDNA-templated formation of copper nanoparticles in solution. Angew. Chem., Int. Ed. Engl..

[9] Song Q., Shi Y., He D., Xu S., Ouyang J. (2015). Sequence-dependent
dsDNA-templated formation of fluorescent copper nanoparticles. Chemistry.

[10] Pang J., Lu Y., Gao X., He L., Sun J., Yang F., Hao Z., Liu Y. (2019). DNA-templated
copper nanoclusters as a fluorescent
probe for fluoride by using aluminum ions as a bridge. Mikrochim. Acta.

[11] Cao Q., Li J., Wang E. (2019). Recent advances in the synthesis
and application of
copper nanomaterials based on various DNA scaffolds. Biosens. Bioelectron..

[12] Wang X.-P., Yin B.-C., Ye B.-C. (2013). A novel fluorescence
probe of dsDNA-templated
copper nanoclusters for quantitative detection of microRNAs. RSC Adv..

[13] Xu F., Shi H., He X., Wang K., He D., Guo Q., Qing Z., Yan L., Ye X., Li D. (2014). Concatemeric dsDNA-templated
copper nanoparticles strategy with improved
sensitivity and stability based on rolling circle replication and
its application in microRNA detection. Anal.
Chem..

[14] Jia X., Li J., Han L., Ren J., Yang X., Wang E. (2012). DNA-hosted
copper nanoclusters for fluorescent identification of single nucleotide
polymorphisms. ACS Nano.

[15] London M., Gallo E. (2020). Epidermal growth factor
receptor (EGFR) involvement in epithelial-derived
cancers and its current antibody-based immunotherapies. Cell Biol. Int..

[16] Kohno T., Nakaoku T., Tsuta K., Tsuchihara K., Matsumoto S., Yoh K., Goto K. (2015). Beyond ALK-RET,
ROS1
and other oncogene fusions in lung cancer. Transl.
Lung Cancer Res..

[17] Hsu K. H., Ho C. C., Hsia T. C., Tseng J. S., Su K. Y., Wu M. F., Chiu K. L., Yang T. Y., Chen K. C., Ooi H. (2015). Identification of five driver gene mutations in patients
with treatment-naive lung adenocarcinoma in Taiwan. PLoS One.

[18] Okabe T., Okamoto I., Tamura K., Terashima M., Yoshida T., Satoh T., Takada M., Fukuoka M., Nakagawa K. (2007). Differential constitutive activation
of the epidermal
growth factor receptor in non-small cell lung cancer cells bearing
EGFR gene mutation and amplification. Cancer
Res..

[19] Lindeman N. I., Cagle P. T., Beasley M. B., Chitale D. A., Dacic S., Giaccone G., Jenkins R. B., Kwiatkowski D. J., Saldivar J. S., Squire J. (2013). Molecular testing guideline
for selection of lung cancer patients for EGFR and ALK tyrosine kinase
inhibitors: guideline from the College of American Pathologists, International
Association for the Study of Lung Cancer, and Association for Molecular
Pathology. J. Mol. Diagn..

[20] Kim S., Misra A. (2007). SNP genotyping: technologies
and biomedical applications. Annu. Rev. Biomed.
Eng..

[21] Kalendar R., Baidyussen A., Serikbay D., Zotova L., Khassanova G., Kuzbakova M., Jatayev S., Hu Y. G., Schramm C., Anderson P. A. (2022). Modified ″Allele-Specific qPCR″
Method for SNP Genotyping Based on FRET. Front.
Plant Sci..

[22] Wang C. C., Chang J. G., Ferrance J., Chen H. Y., You C. Y., Chang Y. F., Jong Y. J., Wu S. M., Yeh C. H. (2008). Quantification
of SMN1 and SMN2 genes by capillary electrophoresis for diagnosis
of spinal muscular atrophy. Electrophoresis.

[23] Ye J., Parra E. J., Sosnoski D. M., Hiester K., Underhill P. A., Shriver M. D. (2002). Melting curve SNP (McSNP) genotyping: a useful approach
for diallelic genotyping in forensic science. J. Forensic Sci..

[24] Hashim H. O., Al-Shuhaib M. B. S. (2019). Exploring the Potential and Limitations of PCR-RFLP
and PCR-SSCP for SNP Detection: A Review. J.
Appl. Biotechnol. Rep..

[25] Si Y., Wang X., Su X., Weng Z., Hu Q., Li Q., Fan C., Zhang D. Y., Wang Y., Luo S., Song P. (2024). Extended Enrichment for Ultrasensitive Detection of
Low-Frequency Mutations by Long Blocker Displacement Amplification. Angew. Chem., Int. Ed. Engl..

[26] Liu Q., Jiang Z., Li S., Li Y., Wan Y., Hu Z., Ma S., Zou Z., Yang R. (2025). Nonequilibrium hybridization-driven
CRISPR/Cas adapter with extended energetic penalty for discrimination
of single-nucleotide variants. Nucleic Acids
Res..

[27] Yu H., Han X., Wang W., Zhang Y., Xiang L., Bai D., Zhang L., Weng Z., Lv K., Song L. (2024). Modified Unit-Mediated Strand Displacement Reactions for Direct Detection
of Single Nucleotide Variants in Active Double-Stranded DNA. ACS Nano.

[28] Du Z., Zhu L., Xu W. (2022). Visualization
of copper nanoclusters for SARS-CoV-2
Delta variant detection based on rational primers design. Talanta.

[29] Lai K. P., Liu B. Y., Tseng W. L., Kou H. S., Wang C. C. (2025). Novel Primer
Design for Significantly Reducing Fluorescent Interferences in the
Synthesis of DNA-Templated Copper Nanoclusters for the Detection of
the HLA-B*5801 Gene. ACS Sens..

[30] Kim S., Lee E. S., Cha B. S., Park K. S. (2022). High Fructose Concentration
Increases the Fluorescence Stability of DNA-Templated Copper Nanoclusters
by Several Thousand Times. Nano Lett..

[31] Chen J., Ji X., Tinnefeld P., He Z. (2016). Multifunctional Dumbbell-Shaped DNA-Templated
Selective Formation of Fluorescent Silver Nanoclusters or Copper Nanoparticles
for Sensitive Detection of Biomolecules. ACS
Appl. Mater. Interfaces.

[32] Chen C. A., Wang C. C., Kou H. S., Wu S. M. (2020). Molecular inversion
probe-rolling circle amplification with single-strand poly-T luminescent
copper nanoclusters for fluorescent detection of single-nucleotide
variant of SMN gene in diagnosis of spinal muscular atrophy. Anal. Chim. Acta.

[33] Vázquez-Vázquez C., Bañobre-López M., Mitra A., López-Quintela M. A., Rivas J. (2009). Synthesis
of Small Atomic Copper Clusters in Microemulsions. Langmuir.

[34] Kim S., Kim J. H., Kwon W. Y., Hwang S. H., Cha B. S., Kim J. M., Oh S. S., Park K. S. (2019). Synthesis of DNA-templated
copper nanoparticles with enhanced fluorescence stability for cellular
imaging. Mikrochim. Acta.

[35] Wei H., Therrien C., Blanchard A., Guan S., Zhu Z. (2008). The Fidelity
Index provides a systematic quantitation of star activity of DNA restriction
endonucleases. Nucleic Acids Res..

[36] Gerlinger M., Rowan A. J., Horswell S., Math M., Larkin J., Endesfelder D., Gronroos E., Martinez P., Matthews N., Stewart A. (2012). Intratumor heterogeneity and branched evolution revealed
by multiregion sequencing. N. Engl. J. Med..

[37] Dagogo-Jack I., Shaw A. T. (2018). Tumour heterogeneity and resistance to cancer therapies. Nat. Rev. Clin. Oncol..

[38] Angulo B., Conde E., Suárez-Gauthier A., Plaza C., Martínez R., Redondo P., Izquierdo E., Rubio-Viqueira B., Paz-Ares L., Hidalgo M. (2012). A Comparison
of Mutation Testing Methods in Lung Carcinoma: Direct Sequencing,
Real-time PCR and Immunohistochemistry. PLoS
One.

[39] Cheng Y. W., Stefaniuk C., Jakubowski M. A. (2019). Real-time
PCR and targeted next-generation
sequencing in the detection of low level EGFR mutations: Instructive
case analyses. Respir. Med. Case Rep..

[40] Lynch T. J., Bell D. W., Sordella R., Gurubhagavatula S., Okimoto R. A., Brannigan B. W., Harris P. L., Haserlat S. M., Supko J. G., Haluska F. G. (2004). Activating mutations
in the epidermal growth factor receptor underlying responsiveness
of non-small-cell lung cancer to gefitinib. N. Engl. J. Med..

[41] Wang C. C., Chen C. A., Jong Y. J., Kou H. S. (2018). Specific Gene Capture
Combined with Restriction-Fragment Release for Directly Fluorescent
Genotyping of Single-Nucleotide Polymorphisms in Diagnosing Spinal
Muscular Atrophy. Anal. Chem..

[42] Lai K. P., Huang Q. Y., Lin K. H., Kou H. S., Wang C. C. (2026). Label-free
fluorescent quantification of L858R single-nucleotide variant in EGFR
gene in single-tube magnetic purification system. Talanta.

