# Supplementary material for: The Specific Low-Interference dsDNA Copper Nanoclusters for Visual Fluorescent Detection and Quantification of the EGFR L858R Point Mutation in Whole Single-Tube Magnetic Purification System
Source: Anal Chem. 2026 Apr 27;98(18):13566–75. doi: 10.1021/acs.analchem.6c00089 (PMC13177290; doi:10.1021/acs.analchem.6c00089)
Supplement: Supplementary file 1 [file ac6c00089_si_001.pdf]

# Supporting Information

## The Specific Low-Interference dsDNA Copper Nanoclusters for Visual Fluorescent Detection and Quantification of the *EGFR* L858R Point Mutation in Whole Single-Tube Magnetic Purification System.

Ke-Peng Lai[a], Ravery Sebuyoya[a], Kung-Hung Lin[a,b,c], Hwang-Shang Kou[a] and Chun-Chi Wang [a,d,e*]

[a] School of Pharmacy, College of Pharmacy, Kaohsiung Medical University, Kaohsiung, 807, Taiwan.

[b] Department of Surgery, Zuoying Armed Forces General Hospital, Kaohsiung, 813, Taiwan.

[c] Department of Family Medicine, Zuoying Armed Forces General Hospital, Kaohsiung, 813, Taiwan.

[d] Department of Medical Research, Kaohsiung Medical University Hospital, Kaohsiung, 807, Taiwan.

[e] Drug Development and Value Creation Research Center, Kaohsiung Medical University, Kaohsiung, 807, Taiwan.

**Abstract:**

Existing fluorescence-based techniques for single-nucleotide variation detection are limited by nonspecific fluorescence interference and complex analytical workflows. To address these challenges, we developed a single-tube fluorescence detection strategy integrating restriction fragment length polymorphism with poly-AAT–templated copper nanoclusters. Owing to the significantly higher synthesis efficiency of copper nanoclusters on AT-rich sequences than on random DNA templates, fluorescence interference from residual genomic DNA and nonspecific amplification products was effectively suppressed. As a result, strong fluorescence emission was generated predominantly from double-stranded poly-AAT, enabling reliable visual discrimination of single-nucleotide variation ratios under UV illumination. Biotin-labeled primers combined with streptavidin-coated magnetic beads enabled efficient separation of digested DNA frag-ments, eliminating the need for electrophoresis and further simplifying the workflow. The entire analytical procedure—including PCR amplification, enzymatic digestion, magnetic separation, and fluorescence measurement—was completed within a single tube, highlighting its suitability for automation and integration into microfluidic platforms. The method was validated for detection of the *EGFR* L858R mutation in patients with non-small cell lung cancer, yielding an excellent linear calibration curve (r = 0.9981), recovery rates of 95–110%, and a detection limit of 2.33%. These results demon-strate sensitivity comparable to that of commercial qPCR and next-generation sequencing, while offering advantages in instrument cost, simplicity, and analytical specificity.

# Contents

**Table S1.** The sequence of primers and templates used in this study.

**Figure S1.** Analysis results of fluorescent wavelength scans using four different primers: (A) Primers labeled with poly-AT sequence; (B) Primers labeled with poly-AAT sequence; (C) Primers labeled with poly-T sequence; and (D) Primers labeled with random sequence.

**Figure S2.** Effect of increasing the number of PCR cycles from 35 to 45 on DNA amplification outcomes. (A) Fluorescence emission spectra corresponding to 35 cycles of wild-type and L858R templates, as well as 45 cycles of wild-type and L858R templates. (B) Capillary gel electrophoresis electropherograms depicting 35 cycles of blank and gDNA samples, 45 cycles of blank and gDNA samples, alongside a 100-bp DNA ladder.

**Figure S3.** Real sample analysis in ten different individuals. (A) The fluorescence intensity of 0 and 100% mutation DNA template (gray bar) compared with pure and spiked genomic DNA samples from ten individuals (black bar). (B) Sanger sequencing results of the ten individuals' gDNA.

**Figure S4.** Fluorescence emission spectra evaluating the analytical specificity of the proposed assay. The spectra represent the fluorescence intensities of the blank sample (black line), *EGFR* L858R template (red line), *EGFR* L861Q template (green line), and *EGFR* wild template (blue line).

**Figure S5.** A comparative evaluation of various DNA fluorescence detection methods, along with their corresponding fluorescence emission spectra. (A) The use of FAM-labeled primer. (B) The use of DNA-binding dye. (C) The use of poly-AAT templated copper nanoclusters (CuNCs).

**Figure S6.** Fluorescence intensity of poly-AAT-templated copper nanoclusters spiked with 0, 50, 100, 150, and 200 ng of genomic DNA. (The total amount of DNA fragments in the 70 μL supernatant was determined using a Qubit™ 4 Fluorometer. L858R DNA template: 92.4 ng; Wild DNA template: 465.1 ng.)

**Table S1.** The sequence of primers and templates used in this study.

| Primer | Sequence (5’→3’) |
|---|---|
| *EGFR*-48AT | ATATATATATATATATATATATATATATATATATATATATATATATATCCCAAA GCAGCTCTGGCTCAC |
| *EGFR*-48AAT | AATAATAATAATAATAATAATAATAATAATAATAATAATAATAATAATCCCAAA GCAGCTCTGGCTCAC |
| *EGFR*-48T | TTTTTTTTTTTTTTTTTTTTTTTTTTTTTTTTTTTTTTTTTTTTTTTTCCCAAA GCAGCTCTGGCTCAC |
| *EGFR*-48Random | GTAGTCGTGATGAACGTATGAGCGTATGAGTATACTCATACGCTCATACCCAAA GCAGCTCTGGCTCAC |
| *EGFR*-Forward primer | Biotin-TGTCAAGATCACAGATTTTGGGC |
| **Template** | **Sequence (5’→3’)** |
| Wild type | AGCAGGGTCTTCTCTGTTTCAGGGCATGAACTACTTGGAGGACCGTCGCTTGGTGCACCG CGACCTGGCAGCCAGGAACGTACTGGTGAAAACACCGCAGCATGTCAAGATCACAGATTT TGGGCTGGCCAAACTGCTGGGTGCGGAAGAGAAAGAATACCATGCAGAAGGAGGCAAAGT AAGGAGGTGGCTTTAGGTCAGCCAGCATTTTCCTGACACCAGGGACCAGGCTGCCTTCCC ACTAGCTGTATTGTTTAACACATGCAGGGGAGGATGCTCTCCAGACATTCTGGGTGAGCT CGCAGCAGCTGCTGCTGGCAGCTGGGTCCAGCCAGGGTCTCCTGGTAGTGTGAGCCAGAG CTGCTTTGGGAACAGTACTTGCTGGGACAGTGAATGAGGATGTTATCCCCAGGTGATCAT TAGCAAATGTTAGGTTTCAGTCTCTCCCTGCAGGATATATAAGTCCCCTTCAATAGCGCA |
| *EGFR* L858R | AGCAGGGTCTTCTCTGTTTCAGGGCATGAACTACTTGGAGGACCGTCGCTTGGTGCACCG CGACCTGGCAGCCAGGAACGTACTGGTGAAAACACCGCAGCATGTCAAGATCACAGATTT TGGGCGGGCCAAACTGCTGGGTGCGGAAGAGAAAGAATACCATGCAGAAGGAGGCAAAGT AAGGAGGTGGCTTTAGGTCAGCCAGCATTTTCCTGACACCAGGGACCAGGCTGCCTTCCC ACTAGCTGTATTGTTTAACACATGCAGGGGAGGATGCTCTCCAGACATTCTGGGTGAGCT CGCAGCAGCTGCTGCTGGCAGCTGGGTCCAGCCAGGGTCTCCTGGTAGTGTGAGCCAGAG CTGCTTTGGGAACAGTACTTGCTGGGACAGTGAATGAGGATGTTATCCCCAGGTGATCAT TAGCAAATGTTAGGTTTCAGTCTCTCCCTGCAGGATATATAAGTCCCCTTCAATAGCGCA |
| *EGFR* L861Q | AGCAGGGTCTTCTCTGTTTCAGGGCATGAACTACTTGGAGGACCGTCGCTTGGTGCACCG CGACCTGGCAGCCAGGAACGTACTGGTGAAAACACCGCAGCATGTCAAGATCACAGATTT TGGGCTGGCCAAACAGCTGGGTGCGGAAGAGAAAGAATACCATGCAGAAGGAGGCAAAGT AAGGAGGTGGCTTTAGGTCAGCCAGCATTTTCCTGACACCAGGGACCAGGCTGCCTTCCC ACTAGCTGTATTGTTTAACACATGCAGGGGAGGATGCTCTCCAGACATTCTGGGTGAGCT CGCAGCAGCTGCTGCTGGCAGCTGGGTCCAGCCAGGGTCTCCTGGTAGTGTGAGCCAGAG CTGCTTTGGGAACAGTACTTGCTGGGACAGTGAATGAGGATGTTATCCCCAGGTGATCAT TAGCAAATGTTAGGTTTCAGTCTCTCCCTGCAGGATATATAAGTCCCCTTCAATAGCGCA |

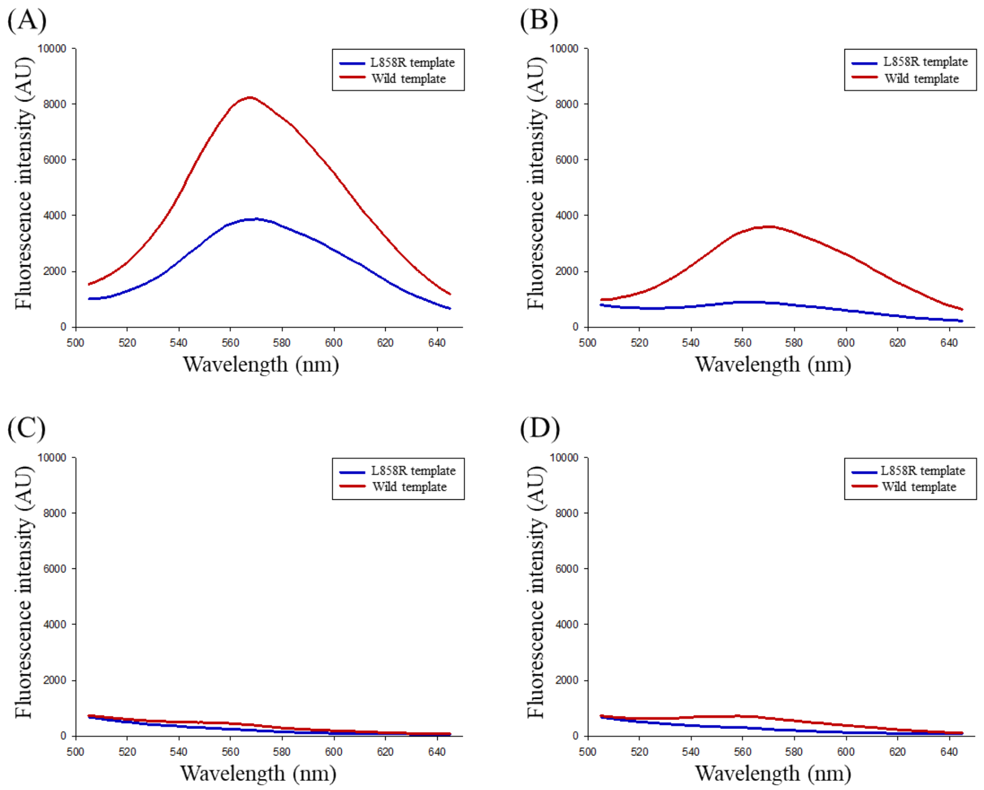


**Figure S1.** Analysis results of fluorescent wavelength scans using four different primers: (A) Primers labeled with poly-AT sequence; (B) Primers labeled with poly-AAT sequence; (C) Primers labeled with poly-T sequence; and (D) Primers labeled with random sequence.

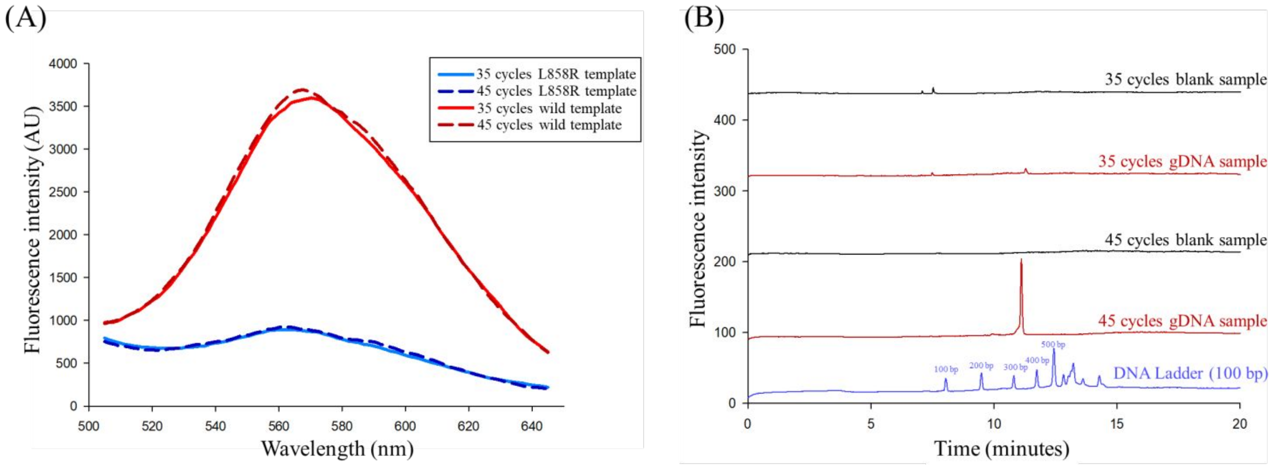


**Figure S2.** Effect of increasing the number of PCR cycles from 35 to 45 on DNA amplification outcomes. (A) Fluorescence emission spectra corresponding to 35 cycles of wild-type and L858R templates, as well as 45 cycles of wild-type and L858R templates. (B) Capillary gel electrophoresis electropherograms depicting 35 cycles of blank and gDNA samples, 45 cycles of blank and gDNA samples, alongside a 100-bp DNA ladder.

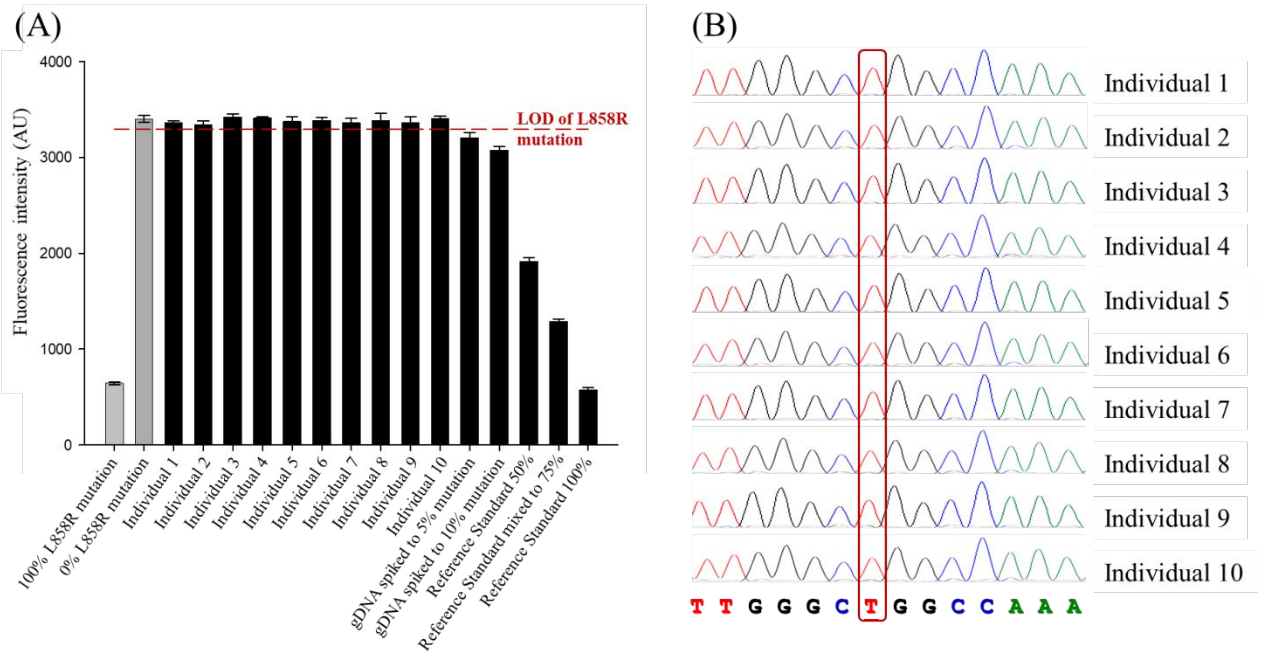


**Figure S3.** Real sample analysis in ten different individuals. (A) The fluorescence intensity of 0 and 100% mutation DNA template (gray bar) compared with pure and spiked genomic DNA samples from ten individuals (black bar). (B) Sanger sequencing results of the ten individuals' gDNA.

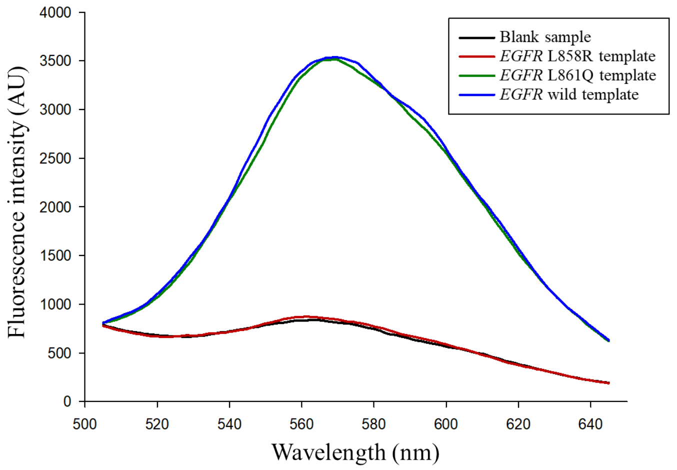


**Figure S4.** Fluorescence emission spectra evaluating the analytical specificity of the proposed assay. The spectra represent the fluorescence intensities of the blank sample (black line), *EGFR* L858R template (red line), *EGFR* L861Q template (green line), and *EGFR* wild template (blue line).

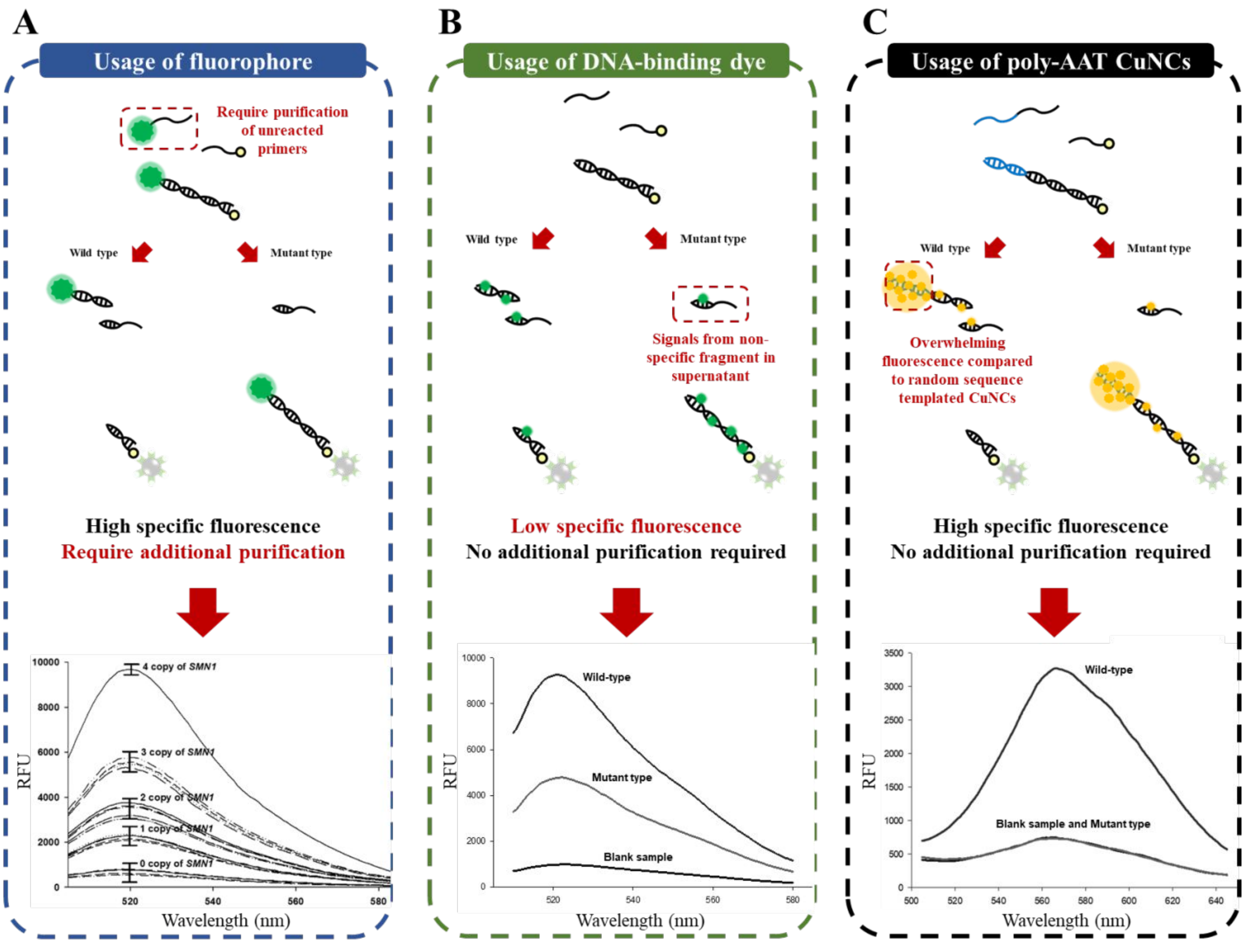


**Figure S5.** A comparative evaluation of various DNA fluorescence detection methods, along with their corresponding fluorescence emission spectra. (A) The use of FAM-labeled primer. (B) The use of DNA-binding dye. (C) The use of poly-AAT templated copper nanoclusters (CuNCs).

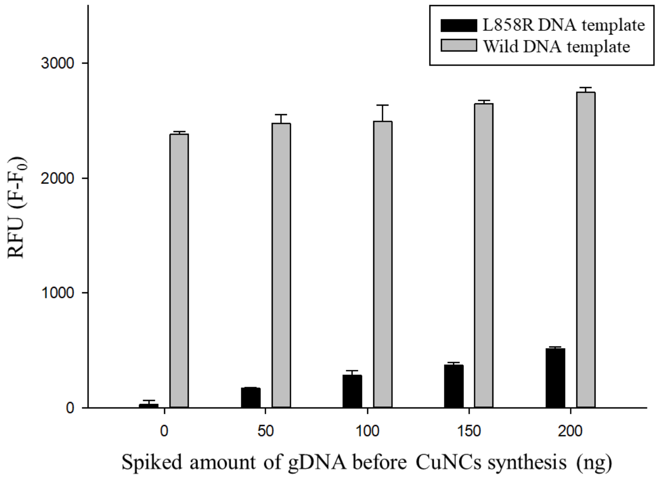


**Figure S6.** Fluorescence intensity of poly-AAT-templated copper nanoclusters spiked with 0, 50, 100, 150, and 200 ng of genomic DNA. (The total amount of DNA fragments in the 70 μL supernatant was determined using a Qubit™ 4 Fluorometer. L858R DNA template: 92.4 ng; Wild DNA template: 465.1 ng.)
